# Etiology and prognosis of pregnancy-related pelvic girdle pain; design of a longitudinal study

**DOI:** 10.1186/1471-2458-5-1

**Published:** 2005-01-03

**Authors:** Janneke M Bastiaanssen, Rob A de Bie, Caroline HG Bastiaenen, Annie Heuts, Mariëlle EAL Kroese, Gerard GM Essed, Piet A van den Brandt

**Affiliations:** 1 Department of Epidemiology, Maastricht University, P.O. Box 616, 6200 MD Maastricht, The Netherlands; 2 Midwifery care, Meerssen, The Netherlands; 3 Department of Integrated Care, University Hospital Maastricht, Maastricht, The Netherlands; 4 Department of Gynaecology, University Hospital Maastricht, Maastricht, The Netherlands

## Abstract

**Background:**

Absence of knowledge of pregnancy-related pelvic girdle pain (PPGP) has prompted the start of a large cohort study in the Netherlands. The objective of this study was to investigate the prevalence and incidence of PPGP, to identify risk factors involved in the onset and to determine the prognosis of pregnancy-related pelvic girdle pain.

**Methods/design:**

7,526 pregnant women of the southeast of the Netherlands participated in a prospective cohort study. During a 2-year period, they were recruited by midwives and gynecologists at 14 weeks of pregnancy. Participants completed a questionnaire at baseline, at 30 weeks of pregnancy, at 2 weeks after delivery, at 6 months after delivery and at 1 year after delivery. The study uses extensive questionnaires with questions ranging from physical complaints, limitations in activities, restriction in participation, work situation, demographics, lifestyle, pregnancy-related factors and psychosocial factors.

**Discussion:**

This large-scale prospective cohort study will provide reliable insights in incidence, prevalence and factors related to etiology and prognosis of pregnancy-related pelvic girdle pain.

## Background

In the Netherlands, little information is available about prevalence, incidence, etiology and prognosis of pregnancy-related pelvic girdle pain (PPGP). It is hypothesized that during pregnancy many women (about 80%) experience some degree of pain in the pelvic region and/or the low back and that in some of these patients pain becomes chronic or recurrent. Often, symptoms impact on activities of daily life, hobbies, participation in society, planning of next pregnancies and sometimes lead to a chronic disabling condition with considerable work absenteeism in the future [1].

Treatment and indirect costs of these chronic or recurrent patients constitute a considerable burden on health care services, health care insurers and other parties. The total health care expenditures incurred by patients with back pain in the United States in 1998 were approximately $91 billion, accounting for about 1 % of the Gross National Product [2]. Van Tulder et al. estimated the costs of back pain for Dutch society in 1991 at 1.7% of the Gross National Product [3]. These costs consist almost totally of indirect costs (97%) such as absenteeism and disablement; for that reason chronic back pain can be considered a major economical problem. It is therefore important that PPGP can be diagnosed and treated before PPGP becomes chronic. Consequently, tracking risk factors and characteristics influencing etiology and prognosis of pregnancy-related pelvic girdle pain is important. Absence of knowledge of risk and prognostic factors and the absence of evidence-based treatment strategies about PPGP has prompted the start of a large cohort study in the Netherlands.

In January 2000, the Maastricht PPGP cohort study started. It was established (I) to examine the prevalence and incidence of pregnancy-related pelvic girdle pain (PPGP) during and after pregnancy, (II) to identify risk factors involved in the onset of PPGP and to identify which factors can play an important role in the early detection of PPGP and finally (III) to determine the prognosis of PPGP and to identify prognostic factors. Furthermore, a clinical trial is embedded in this cohort, aimed at studying the effectiveness of a tailor-made treatment program in PPGP after delivery [Bastiaenen et al., treatment, submitted].

## Methods/design

### Design and study population

In an observational prospective cohort study, etiology and prognosis of PPGP will be studied in 7526 pregnant women. The source population for the study comprises of pregnant women from the southeastern area of the Netherlands.

Both midwives and gynecologists recruit women for the study when pregnant between 10 and 14 weeks. Women are considered eligible if they meet the following inclusion criteria: women are well versed in the Dutch language and at least 18 years. At inclusion, women receive a leaflet containing information about the research project, an informed consent form and a baseline questionnaire. After providing informed consent and filling out the first questionnaire, the women receive a second questionnaire at 30 weeks of pregnancy, a third one at 2 weeks after delivery and a fourth and fifth at 6 months and 12 months postpartum, respectively.

In this study, extensive questionnaires are used with questions ranging from physical complaints (past and present), limitations in activities, restriction in participation, work situation, demographics, lifestyle, pregnancy-related factors and psychosocial factors. With the help of these questionnaires the prevalence and incidence of PPGP in the Netherlands will be described. In addition, possible risk factors and prognostic factors of PPGP will be examined.

### Exposure variables

In the Maastricht PPGP cohort study several domains of exposure were measured, including individual characteristics, lifestyle, work situation, pregnancy-related factors and psychosocial factors. The majority of factors were assessed with existing, validated questionnaires. The Dutch translation of the Quebec Back Pain Disability Scale (QBPDS) [4] will measure low back functional status [5]. The QBPDS is a 20-item 6-point scale describing activities commonly affected by back pain. This questionnaire is not developed to study a pregnant population and some activities were unsuitable for women who were pregnant or just gave birth. We therefore added a 7^th ^option to the questionnaire, namely "not applicable". We also changed the phrase "because of my back " into "because of my back and/or pelvic pain" in questionnaires. Fear of movement is measured by means of the Dutch translation of the Tampa Scale for Kinesiophobia (TSK) [6,7]. The TSK consists of 17 items; each rated on a 4-point Likert scale. Pain catastrophizing is measured by the Pain Catastrophizing Scale (PCS) [8]. The PCS is a 13-item 5-point scale. A woman is said to catastrophize pain, when she views pain as extremely threatening. To measure the experience of negative affect and positive affect we used the 14-item Negative Emotionality Scale (NEM) and the 11-item Positive Emotionality Scale (PEM) [9].

Current mental health was measured by the General Health questionnaire (GHQ). The questionnaire was originally designed as a 60-item instrument, but we used the shortened version GHQ-12 [10].

The perceived stress scale (PSS) was used to measure to assess stress. The PSS is a 14-item instrument with a 5-point scale [11].

### Outcome measurements

Pregnancy-related pelvic girdle pain is currently not an entity that can be clearly diagnosed and described. Therefore, Bastiaenen et al. studied separate diagnostic strategies of four international authors in the field of PPGP [Bastiaenen et al., submitted]. They concluded that there was no similarity in the selection of patients with PPGP between the authors. Most of these classification-strategies of PPGP are based on expert-opinions. Therefore, a possible reason for the lack of similarity in the selection of patients can be that they all select different small parts of the same large patient-group.

Because of the relatively unknown etiology of pregnancy-related pelvic girdle pain and the lack of an all-embracing definition, we will use an extensive description of PPGP. We expect that during pregnancy almost all women experience some form of pain in the lower back, the buttocks, the symphyses, the groins and/or radiation into the legs. This pain is probably caused by hormonal and physiological changes which are considered normal during pregnancy. However, some women experience pain in a very early stage of pregnancy while others only experience pain in the final stages of pregnancy. In addition, some women are more limited in their activities (due to pain) than others. This suggests that other factors might influence the hormonal or physiological changes during pregnancy [12]. Most women who had developed PPGP during pregnancy quickly recover after delivery [13].

In this study, pain during or after pregnancy is measured by using patients' self-reports. Women with pain can be identified by the question whether they experienced pain in the lower back, the buttocks, the symphyses, the groins or radiation into the legs during or after this pregnancy. To study etiology of PPGP, women who gave a positive answer to this question during this pregnancy were selected.

**Figure 1:**
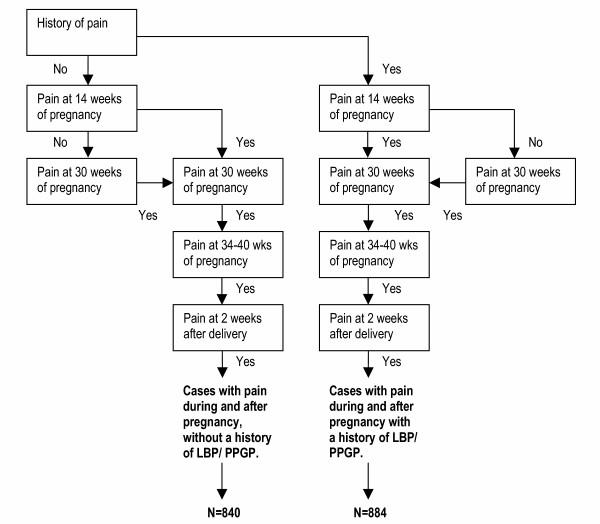
Flowchart of pain in the lower back, the buttocks, the symphyses, groins and/or radiation into the legs: description of cases.

For the prognosis of PPGP it is important that women have pain that started during pregnancy and persisted after delivery. At several moments during and after pregnancy, experienced pain in the lower back, the buttocks, symphyses, groins or radiation into the legs was measured. The answers to this question were assimilated into a flowchart (Fig. 1).

Based on their self-reports, the women are stratified into a case group without a history of LBP/PPGP or a case group with a history of LBP/PPGP. Some women (N = 246) were not classified into groups for the following reasons; specific disorders of the spine, rheumatism, neurological disorders and cancer. The first group consists of women without a history of LBP/PPGP, but they experience pain during pregnancy and this pain does not disappear until (at least) 2 weeks after delivery. The second group experiences pain during and after pregnancy, but they also have a history of LBP/PPGP. Both groups of cases will be analysed to study the prognosis of PPGP.

Women who experience recurrent pain episodes during pregnancy, that resolve within 2 weeks after delivery, will not be considered cases in the analyses for the prognosis of PPGP. They form a miscellaneous group. However, this miscellaneous group and women who experience no pain after delivery were not excluded from follow-up.

### Data analyses

Participation rates and descriptions of baseline characteristics of the Maastricht PPGP cohort study will be presented. We calculated prevalence rates for PPGP by dividing the numbers of prevalent cases at several moments during pregnancy by the total number of subjects.

### Characteristics of the study population at baseline

**Figure 2:**
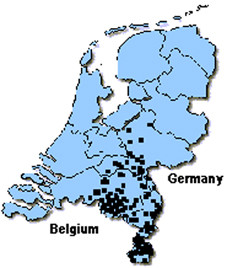
The location of participating midwives and gynaecologists in the Netherlands.

Recruitment of women into the study began in November 2000 and ended in November 2002. Approximately 10,850 women were asked to participate in the study by midwives and gynecologist in the southeast of the Netherlands. The locations of the participating midwives and gynecologists are shown in Fig. 2. At the end of the recruitment period 7,526 pregnant women (73.4%) were willing to participate and were included in the cohort.

In Table 1 a number of selected characteristics of the study population at baseline are presented, including details of their age, education, BMI, smoking, work and reproductive history. Mean age of the study population is 31.5 years. The educational level of the participants is very high. Approximately 38 % of the participants have had higher vocational or academic education. The use of alcohol during pregnancy is limited. While 44.8% of the study population did not use alcohol before pregnancy, 91.2% did not use alcohol during pregnancy. With exception of the country of birth, the study population is heterogeneous with respect to demographics, work status and pregnancy-related factors.

**Table 1:** Characteristics of the study population (N = 7526) at baseline (14 wks pregnancy)

**Aspect**				%
Age (yrs)	Mean		31.54	
N = 7523	< = 20			0.4
	21–25			5.3
	26–30			32.4
	31–35			47.8
	36–40			12.8
	>40			1.2

Country of origin	Netherlands			96.5
N = 7516	Belgium, Germany, France, Austria, Switzerland, Luxemburg, Ireland and United Kingdom			1.9
	Other countries			1.6

Education	Primary school			0.6
N = 7498	Preparatory vocational education			7.2
	Lower general secondary education			6.6
	Vocational education			31.9
	Higher general secondary education			8.5
	Pre-university education			2.3
	Higher vocational education			27.7
	Academic education			10.3
	Different			4.9

BMI before pregnancy	< 18.5	Underweight		3,1
N = 7437	18.5–24.9	Normal weight		68,1
	25.0–29.9	Overweight		20,5
	> = 30	Extreme overweight		8,3

Smoking habits at 14 wks pregnancy	Never			59.7
N = 7488	Ex			29.8
	Current during pregnancy			10.5

Alcohol-usage (glasses/week) before pregnancy	0			44.8
N = 7482	1–10			53.5
	11–20			1.6
	>20			0.2

Alcohol-usage (glasses/week) during pregnancy	0			91.2
N = 7210	1–10			8.8
	11–20			0.0
	>20			0.0

Work (hours/week)	No job			13.9
N = 7428	1–10			3.8
	11–20			22.9
	21–30			21.1
	31–40			36.9
	>40			1.5

Number of pregnancies	1			42.3
N = 7519	2			36.6
	3			14.2
	4			4.6
	>4			2.3

To examine whether the response in our study affected the determinant distributions (e.g. did primarily women in their first pregnancy respond?), a comparison of response rates was carried out. We performed a pilot-study in which data was recorded from April 2001 until November 2001 of every pregnant woman who attended one of the cooperating practices. Information about parity, PPGP during pregnancy, delivery and PPGP after delivery (until 6 weeks) was collected of 283 women (170 participants and 113 non-participants). Results of this pilot-study showed that primipara compared to multiparous women were more willing to participate in the cohort study.

Furthermore, data from the responders in the cohort was compared to available data on pregnant Women in The Netherlands from the "Centraal Bureau voor Statistiek". The results of this comparison are shown in table 2.

**Table 2:** A comparison between the total Dutch female population and the study population

	**Total Dutch population**			**Study population**		
	Period 2001	Period 2002	Period 2003	Period 2001 (N = 2892)	Period 2002 (N = 3567)	Period 2003 (N = 1061)

BMI	%*	%*	%*	%	%	%
- Underweight (<18.5)	5.3	4.9	3.0	2,9	3,2	3,5
- Normal weight (18.5–24.9)	70.3	71.9	71.2	66,8	68,5	70,4
- Overweight (25.0–29.9)	18.5	17.8	18.0	21,3	20,3	18,9
- Extreme overweight (> = 30)	5.9	5.4	7.8	9,1	8,1	7,2

Smoking	%*	%*	%*	%	%	%
- Current/before pregnancy	35.7	34.0	33.3	29.7	29.3	31.7

**Pregnancy-related factors**						

Mean age mother						
- Total pregnancies	30.8	30.9	31.0	31.1	31.7	32.4
- First pregnancies	29.2	29.2	29.3	29.6	30.2	30.9

Child	%	%	%	%	%	%
- Boy	51.2	51.3	51.4	51.0	52.2	50.5
- Girl	48.8	48.7	48.6	49.0	47.8	49.5

Number of pregnancies:	%	%	%	%	%	%
- First	46.3	45.8	45.5	44,6	41,0	41.0
- Second	36.3	36.7	36.9	35,6	37,2	37.3
- Third	12.3	12.5	12.6	13,6	14,7	13.9
- Fourth or more	5.1	5.0	5.0	6,2	7,1	7.8

Multiple pregnancy	%	%	%	%	%	%
- Twin	1.9	1.9	1.8	1.0	0.9	1.1

* Age-group comparable to study-population

Data are presented in 3 separate years to show possible fluctuations. It is noticeable that the mean age of pregnant women is increasing during the 3-year period in the study population and in the total Dutch population. In the study population, pregnant women are slightly older and heavier compared to the total Dutch population. Approximately 34% of all Dutch women (age 18–45 years), compared to 30% in the study population, are smoking (see table 2). During pregnancy, 10.5% of the study population will continue smoking (see table 1). In general, data of the study population correspond with data of the total Dutch population.

### Prevalence of pregnancy-related pelvic girdle pain during pregnancy

**Figure 3:**
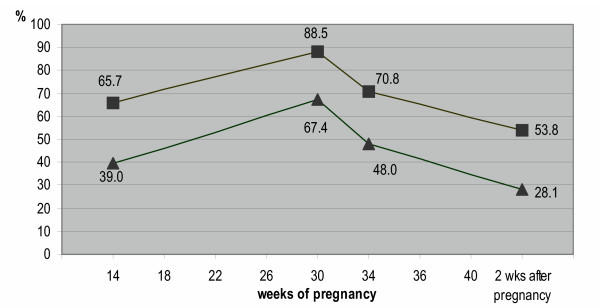
The point prevalence of PPGP during pregnancy for women with (■) and without (▲) a history of LBP/PPGP.

To determine the prevalence of PPGP during pregnancy, data at baseline (14 wks), 30 weeks of pregnancy and 2 weeks after delivery (information about 34–40 weeks of pregnancy) were used. Almost every woman develops pain in the lower back, the buttocks, the symphyses, the groins or radiation into the legs at some time in their pregnancy. Of the 7527 women, 84% reported pain in any or all of these areas during pregnancy. Women with a history of LBP/PPGP are more likely to develop PPGP during pregnancy then women without a history of LBP/PPGP (see fig. 3).

## Discussion

In this article, we describe the main characteristics of the Maastricht PPGP cohort study in terms of study design, study population, exposure variables and outcome measures. This design, in which both risk factors and outcomes are frequently measured between 14 weeks of pregnancy and 1 year after delivery, enables us to examine the etiology and prognosis of PPGP.

### Advantages and disadvantages of the study

Although a cohort design (even incorporating a randomized controlled trial) has advantages over other epidemiological designs, it poses also a burden on the project in terms of logistics and recruitment. First of all, we are totally dependent on the cooperation and recruiting power of midwives and gynecologists. The number of pregnant women is about evenly divided between the two professions. Although professional workload for midwives in the Netherlands is extremely high, 62 % of the midwife practices took part in this study, recruiting about 90% of the patients. Whereas 58% of the hospitals in the recruitment area participate, they only recruit about 10% of the women. Coordination of the recruitment and frequent staff changes in the hospitals themselves seems to be a key problem.

In etiological research there should be sufficient contrast in exposure. In this study, the study population at baseline is heterogeneous with respect to demographic variables, lifestyle characteristics and work-related factors. However, due to logistic constraints we have restricted the recruiting area to the southeast of the Netherlands, posing questions about the representation of our sample for the whole Dutch population. For instance the number of immigrants is significantly less in the southeast of the Netherlands. However, for future genetically oriented studies, this could be a major advantage. We have shown previously that our sample is slightly different from the national population of pregnant women with regards to several determinants (e.g. age, BMI).

At baseline, 7526 pregnant women participated in our study. This is a response rate of 73.4%. Non-response and loss to follow-up might introduce selection bias in prospective studies. Loss to follow-up, especially in time series designs with repeated measurements, pose threats to representation of the sample. Especially when losses-to-follow-up are connected with negative pregnancy outcomes (miscarriage, birth defects), co-morbidity of the mother or factors predicting for PPGP. To evaluate whether differential loss to follow-up occurs, we will compare the profile of those lost to follow-up with other participants.

In this study we were able to evaluate if there are significant differences between participants and non-participants. This evaluations shows that primiparous women are more willing to participate in the study compared to multiparous women. We expect that multiparity plays an important role in the etiology and prognosis of PPGP. Therefore, an underestimation of the prevalence of PPGP reported in this study cannot be ruled out. However, the collected sample should be able to provide reliable preliminary insights in incidence and prevalence of PPGP and factors related to etiology and prognosis of PPGP in the Netherlands.

PPGP is a complex syndrome and for a greater understanding of pregnancy-related pelvic girdle pain, future studies should further disentangle the multifactorial etiology and prognosis of PPGP. Future research within the framework of the Maastricht PPGP cohort study will focus on disentanglement of the complex syndrome.

## Competing interests

The author(s) declare that they have no competing interests.

## Authors' contributions

All authors participated in the design of the study. JMB drafted the manuscript with input from the other authors. All authors read, revised and approved the final manuscript.

## Pre-publication history

The pre-publication history for this paper can be accessed here:


